# Dimethyl Fumarate and Cobalt Protoporphyrin IX Inhibit Fibromyalgia-like Pain by Modulating Region-Specific Redox Pathways

**DOI:** 10.3390/antiox15091080

**Published:** 2026-08-28

**Authors:** Sylmara Esther Negrini-Ferrari, Weitao Wang, Olga Pol

**Affiliations:** 1 Grup de Neurofarmacologia Molecular, Institut de Recerca Sant Pau (IR SANT PAU), 08041 Barcelona, Spain; 2 Grup de Neurofarmacologia Molecular, Institut de Neurociències, Universitat Autònoma de Barcelona, 08193 Barcelona, Spain

**Keywords:** fibromyalgia, nociplastic pain, dimethyl fumarate, cobalt protoporphyrin IX, NRF2, HO-1, oxidative stress, NLRP3 inflammasome, depression, neuroplasticity

## Abstract

Fibromyalgia is a chronic nociplastic pain disorder characterized by widespread pain and affective disturbances, in which oxidative stress and neuroinflammation are now recognized as central pathogenic mechanisms. However, current therapies provide only limited symptomatic relief. This study compared the therapeutic and molecular effects of dimethyl fumarate (DMF), an activator of nuclear factor erythroid 2-related factor 2 (NRF2), and cobalt protoporphyrin IX (CoPP), an inducer of heme oxygenase-1 (HO-1), in a reserpine-induced mouse model of fibromyalgia-like pain. Male and female C57BL/6J mice received repeated reserpine administration to induce mechanical allodynia, thermal hyperalgesia, cold allodynia, and depressive-like behaviors. Therapeutic efficacy was evaluated by behavioral testing and molecular analyses of the periaqueductal gray (PAG), anterior cingulate cortex (ACC), and spinal cord (SC). DMF produced a faster antinociceptive response than CoPP, although both compounds ultimately reversed nociceptive hypersensitivity and depressive-like behaviors in male and female mice. These behavioral alterations were associated with increased NLRP3 expression and activated AKT signaling, together with region-specific redox dysregulation involving differential regulation of NADPH oxidases and endogenous antioxidant defenses. Both treatments differentially modulated region-specific redox alterations, enhanced selected antioxidant defenses, and attenuated inflammatory and nociception-related signaling. These findings identify region-specific redox dysregulation as a prominent feature of fibromyalgia-like pathology, providing new mechanistic insight into the molecular basis of nociplastic pain. Collectively, these results demonstrate that DMF and CoPP treatments remodel region-specific redox networks while inhibiting both sensory and affective manifestations of fibromyalgia-like pain, supporting endogenous antioxidant pathways as promising mechanism-based therapeutic targets for fibromyalgia.

## 1. Introduction

Fibromyalgia is a chronic nociplastic disorder characterized by widespread musculoskeletal pain accompanied by fatigue, sleep disturbances, cognitive dysfunction, and affective symptoms [1]. It affects approximately 2–4% of the population and is markedly more prevalent in women. Fibromyalgia constitutes a major public health problem due to its substantial impact on quality of life, daily functioning, and healthcare utilization [2]. Despite substantial advances in chronic pain research, the biological mechanisms driving the initiation and persistence of fibromyalgia remain only partially understood.

Pharmacological therapy continues to play an important role in the management of fibromyalgia. Current pharmacological treatment mainly relies on gabapentinoids and antidepressants, such as pregabalin, duloxetine, and milnacipran [3]. However, their clinical effectiveness is highly variable, with only a subset of patients achieving clinically meaningful pain relief (commonly defined as ≥30% reduction in pain intensity), while the overall benefits are frequently modest and accompanied by adverse effects [3]. These limitations highlight the need to identify new therapeutic approaches targeting molecular mechanisms involved in fibromyalgia pathophysiology rather than providing only symptomatic control.

Current evidence indicates that fibromyalgia results from a complex interaction between altered nociceptive processing, neuroimmune dysregulation, oxidative stress, mitochondrial dysfunction, and maladaptive neuronal plasticity [1,2,4]. Central sensitization, characterized by enhanced nociceptive transmission and impaired descending inhibitory control, is considered a key mechanism underlying chronic pain in fibromyalgia [1,2]. In addition, elevated levels of inflammatory mediators have been reported in fibromyalgia patients and are associated with symptom severity, supporting an important role for neuroinflammatory signaling in both sensory and affective manifestations of the disorder [5,6,7].

Amongst the inflammatory pathways implicated in chronic pain, the NOD-like receptor protein 3 (NLRP3) inflammasome has emerged as a key molecular regulator linking cellular stress to neuroinflammatory responses. NLRP3 activation promotes the maturation and release of pro-inflammatory cytokines, including IL-1β and IL-18, thereby enhancing neuronal excitability and facilitating central sensitization [8,9]. In addition, activation of intracellular signaling pathways involved in neuronal plasticity, particularly AKT signaling, further contributes to the maintenance of persistent pain by promoting maladaptive changes in neuronal activity [10].

Oxidative stress is also increasingly recognized as a major contributor to fibromyalgia pathophysiology [11]. Clinical and experimental studies have demonstrated increased oxidative damage together with impaired antioxidant defenses in fibromyalgia [12,13,14]. Importantly, oxidative stress interacts closely with neuroinflammatory and plasticity-related mechanisms and may facilitate NLRP3 inflammasome activation, neuronal sensitization, and chronic pain maintenance [8,14,15,16]. Among the enzymatic sources of reactive oxygen species (ROS), NADPH oxidases (NOX) have emerged as key contributors to central sensitization and persistent pain [15]. NOX have been implicated in the maintenance of chronic pain through sustained ROS production, which amplifies neuroinflammatory signaling, promotes NLRP3 expression, enhances neuronal plasticity, and contributes to pain chronification [15,16]. Despite these advances, the region-specific contribution of individual NOX isoforms to nociplastic pain and its associated affective disturbances remains poorly understood.

The transcription factor nuclear factor erythroid 2-related factor 2 (NRF2) constitutes the principal endogenous mechanism limiting oxidative damage by coordinately regulating antioxidant defenses and suppressing excessive ROS production from both mitochondria and NOX, thereby preserving cellular redox homeostasis [17]. Upon activation, NRF2 promotes the expression of several antioxidant and cytoprotective enzymes, including heme oxygenase-1 (HO-1), superoxide dismutase-1 (SOD-1), and NAD(P)H quinone oxidoreductase-1 (NQO1). Beyond its antioxidant role, NRF2 negatively regulates inflammatory signaling, suppresses NLRP3 up-regulation and modulates neuronal plasticity [18,19,20,21]. Consequently, pharmacological targeting of the NRF2/HO-1 pathway may represent a promising therapeutic strategy for modulating multiple mechanisms implicated in the development and maintenance of nociplastic pain.

Dimethyl fumarate (DMF) is an orally available NRF2 activator clinically used for the treatment of relapsing-remitting multiple sclerosis and moderate-to-severe plaque psoriasis [21,22]. Besides its established clinical efficacy, DMF activates NRF2-dependent cytoprotective programs, reduces oxidative stress, suppresses neuroinflammatory signaling, and improves mitochondrial function, effects that have also been documented [20,21,23]. Cobalt protoporphyrin IX (CoPP), in turn, is a potent inducer of HO-1 through mechanisms involving activation of antioxidant transcriptional programs [24,25].

Although both compounds and related NRF2/HO-1 modulators have shown beneficial effects in experimental models of inflammatory and neuropathic pain [26,27,28,29], the therapeutic potential of DMF and CoPP in nociplastic pain remains largely unexplored. Moreover, given their distinct pharmacological profiles, comparing DMF and CoPP provides an opportunity to investigate whether upstream activation of endogenous antioxidant defenses produces greater molecular and therapeutic benefits than selective induction of a single downstream effector.

We hypothesized that DMF would produce broader region-specific remodeling of antioxidant, inflammatory, and/or neuronal plasticity pathways than CoPP, thereby potentially leading to faster and more effective inhibition of nociceptive hypersensitivity and/or depressive-like behaviors.

To test this hypothesis, we compared the therapeutic effects of DMF and CoPP in a reserpine-induced mouse model of fibromyalgia-like pain. We evaluated their ability to inhibit nociceptive hypersensitivity and depressive-like behaviors and modulate molecular pathways associated with oxidative stress, neuroinflammation, and neuronal plasticity in the periaqueductal gray (PAG), anterior cingulate cortex (ACC), and/or spinal cord (SC), regions critically involved in pain modulation and affective processing [30,31,32].

## 2. Materials and Methods

### 2.1. Animals

Adult male and female C57BL/6J mice (8–10 weeks old; 21–26 g) were obtained from Envigo (Barcelona, Spain). Animals were maintained under standard laboratory conditions, including a 12 h light/dark cycle, controlled temperature (22 ± 1 °C), and relative humidity (55 ± 10%), with free access to food and water. Mice were housed in groups of four per polypropylene cage containing wood shavings and kept in an enriched environment equipped with a cardboard shelter and cellulose nesting material. In females, the estrous cycle was monitored daily by vaginal cytology, and all behavioral assessments and tissue sampling were carried out during the diestrus phase to reduce hormonal variability.

All experiments were performed following a seven-day acclimatization period and adhered to the ethical standards established by the Directive 2010/63/EU and Spanish regulations (RD 53/2013). Experimental protocols were approved by the Animal Care and Use Committee of the Autonomous University of Barcelona (approval code: 4581; date of approval 31 March 2023). The study was designed and reported in accordance with the ARRIVE 2.0 guidelines. Every effort was made to minimize animal distress and to use the minimum number of animals necessary, in accordance with the principles of the 3Rs (replacement, reduction, refinement). Animals were monitored daily throughout the study for general health and welfare. No unexpected adverse events occurred, no predefined humane endpoints were reached, and no animals required early euthanasia.

Male and female mice were randomly assigned to the different experimental groups using a computer-generated randomization sequence (Microsoft Excel, =RAND ( )). The required sample size was calculated a priori with G*Power (v3.1.7) based on pilot data, assuming a two-tailed test (α = 0.05, β = 0.2). This analysis indicated that a minimum of six animals per group was necessary to detect significant differences in nociceptive and depressive-like outcomes. To comply with the 3Rs principles, tissues collected from animals used for behavioral testing were subsequently processed for molecular analyses.

Behavioral assessments were conducted between 9:00 AM and 5:00 PM. Prior to testing, animals were habituated to the experimental room for 1 h. For each assay, the testing order was randomized daily, and each animal was evaluated at varying times across test days to minimize potential time-related bias. Two independent researchers were involved in the experimental procedures. The primary investigator administered treatments according to the predetermined randomization schedule and was the only individual aware of group allocation. A second investigator, blinded to treatment conditions, performed the evaluation of mechanical allodynia, thermal hyperalgesia, cold allodynia and depressive-like behaviors.

### 2.2. Establishment of a Fibromyalgia-like Condition

Fibromyalgia-like symptoms were induced using a previously validated protocol [33]. Reserpine was first dissolved in acetic acid and subsequently diluted with 0.9% sodium chloride (NaCl) solution to achieve a final acetic acid concentration of 0.5%. This preparation was administered subcutaneously (s.c.) at a dose of 0.25 mg/kg on days 0, 1, 2, 9, 16, and 23. Control animals received s.c. injections of the corresponding vehicle following the same dosing schedule as the reserpine-treated group.

### 2.3. Assessment of Nociceptive Responses

Mechanical nociceptive thresholds were evaluated using the von Frey filament assay (North Coast Medical, San Jose, CA, USA). This technique utilizes a set of calibrated nylon monofilaments with progressively increasing stiffness, each delivering a defined force when applied to the plantar surface of the paw [34]. Briefly, mice were placed individually in transparent Plexiglas chambers (20 cm height × 9 cm diameter) positioned on a metal mesh platform. Filaments ranging from 0.4 to 3.5 g were applied perpendicularly to the plantar surface, starting with the lowest force (0.4 g). Testing was conducted on the hind paws of both vehicle- and reserpine-injected animals. Based on the animal’s response, the applied force was adjusted up or down according to the up–down method. A positive response was considered as paw withdrawal, licking, or shaking. Mechanical withdrawal thresholds were calculated using a curve-fitting approach implemented in a custom spreadsheet developed with Microsoft Excel (Microsoft Iberia SRL, Barcelona, Spain).

Thermal hyperalgesia was evaluated by determining paw withdrawal latency in response to a radiant heat stimulus using the plantar test apparatus (Ugo Basile, Varese, Italy). Mice were individually placed in transparent Plexiglas chambers (20 cm height × 9 cm diameter) on a glass platform and allowed to habituate for 30 min prior to testing. A focused heat stimulus was then directed to the plantar surface of the hind paws. To avoid tissue injury, a cut-off time of 12 s was established when no withdrawal response occurred. Paw withdrawal latency was calculated as the average of three separate measurements obtained at suitable intervals within each session.

Cold sensitivity was evaluated using a cold plate apparatus (Ugo Basile, Varese, Italy). Mice were placed individually on a surface maintained at 4 ± 0.5 °C for 5 min, and cold allodynia was quantified by counting the number of hind paw lifts during the test period.

### 2.4. Assessment of Depressive-like Behaviors

Depressive-like behaviors were evaluated using the tail suspension test (TST) and the forced swimming test (FST).

In the TST, mice were suspended by the tail using adhesive tape placed approximately 1 cm from the tip and secured to a horizontal bar positioned 35 cm above the floor. Each session lasted 8 min, and immobility time was quantified during the final 6 min [35].

In the FST, mice were individually placed in a Plexiglas cylinder (25 cm height × 10 cm diameter) filled with water maintained at 24 ± 1 °C to a depth of 10 cm. Behavior was recorded for 6 min, and immobility time was analyzed during the last 4 min of the test [36].

### 2.5. Drugs

Reserpine, acetic acid, NaCl, DMF, and CoPP were purchased from Sigma-Aldrich (St. Louis, MO, USA). DMF was administered orally (p.o.) at 300 mg/kg once daily, a regimen previously shown to produce antinociceptive effects and activate NRF2-dependent antioxidant signaling in experimental pain models [19,29]. CoPP was administered intraperitoneally (i.p.) at 2.5 mg/kg twice daily, following previous studies demonstrating effective HO-1 induction and antinociceptive activity with this dosing regimen [37]. Thus, the different dosing schedules used for DMF and CoPP were based on previously established effective pharmacological protocols for each compound rather than on a direct pharmacokinetic comparison between the treatments.

All treatments were freshly prepared and administered at a final volume of 10 mL/kg. Control animals received the corresponding vehicle under the same experimental conditions. In accordance with previous reports, behavioral assessments were performed 3 h after treatment administration [18,19,37].

### 2.6. Experimental Protocol

Experiments were conducted in male and female C57BL/6J mice to investigate potential sex-dependent differences in the therapeutic-like effects of DMF and CoPP in a reserpine-induced model of fibromyalgia-like pain.

Mice received repeated administration of reserpine (0.25 mg/kg, s.c.) or vehicle on days 0, 1, 2, 9, 16, and 23. Once nociceptive hypersensitivity was established, animals were treated from day 25 to day 28 with DMF once daily or CoPP twice daily.

**Figure 1:**
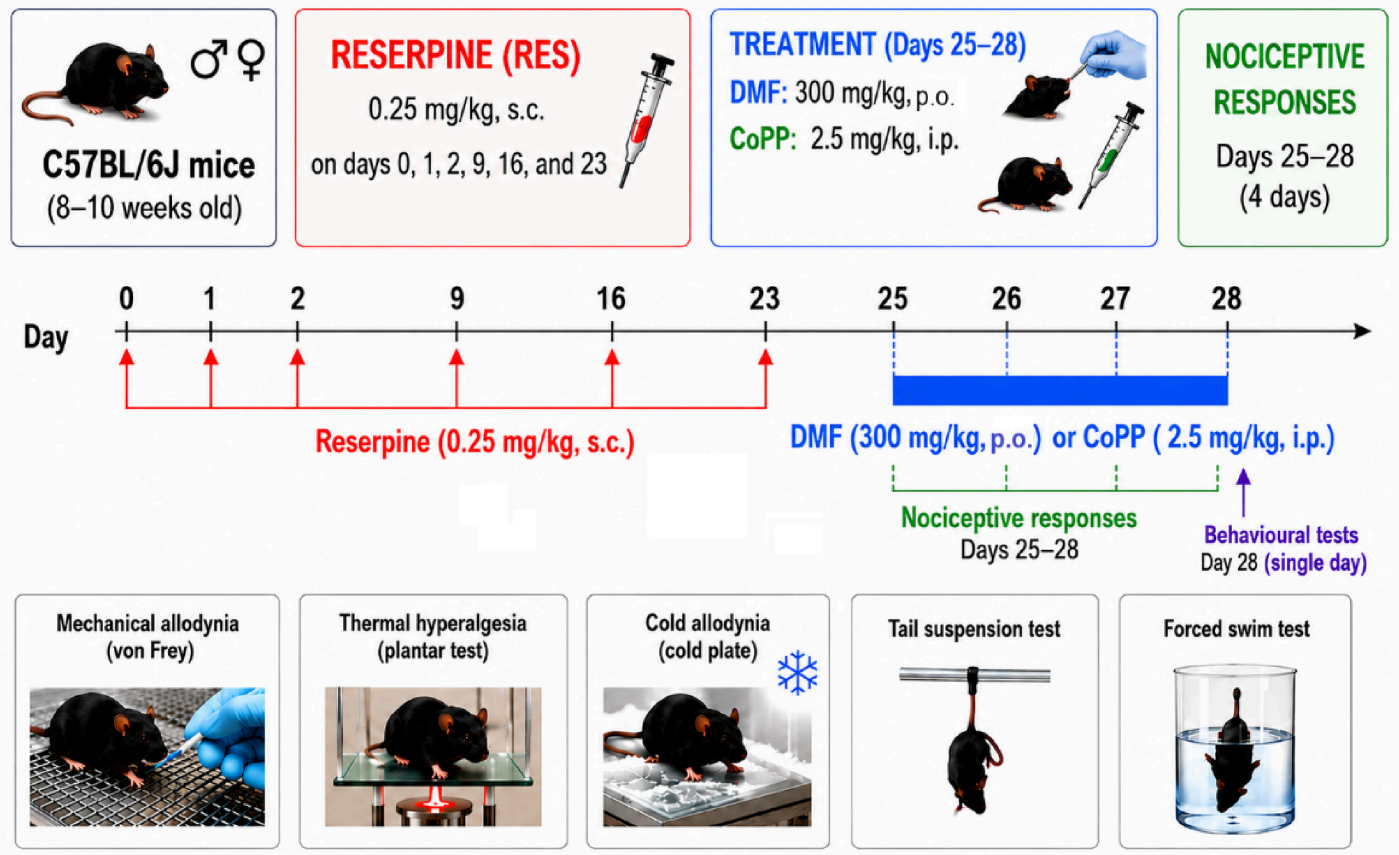
Experimental design of the reserpine-induced nociplastic pain model and pharmacological treatments. Male and female C57BL/6J mice (8–10 weeks old) received reserpine (0.25 mg/kg, s.c.) on days 0, 1, 2, 9, 16, and 23 to induce nociplastic pain and depressive-like behaviors. From day 25 to day 28, animals received dimethyl fumarate (DMF, 300 mg/kg, p.o.) once daily or cobalt protoporphyrin IX (CoPP, 2.5 mg/kg, i.p.) twice daily. Nociceptive responses, including mechanical allodynia (von Frey test), thermal hyperalgesia (plantar test), and cold allodynia (cold plate), were assessed between days 25 and 28. Depressive-like behaviors were evaluated on day 28 using the tail suspension test and the forced swimming test in a separate cohort of animals.

Mechanical allodynia, thermal hyperalgesia, and cold allodynia were evaluated before treatment initiation (day 24) and throughout the treatment period (days 25–28) using the von Frey, plantar, and cold plate tests, respectively, to determine the effects of both compounds on reserpine-induced nociceptive responses (*n* = 6 animals per group; Figure 1).

In an additional independent cohort, the effects of DMF and CoPP on depressive-like behaviors associated with the fibromyalgia-like phenotype were assessed on day 28, after completion of the treatment regimen, using the TST and FST (*n* = 6 animals per group).

Finally, the molecular effects of DMF and CoPP were evaluated in the PAG, ACC and SC of male and female reserpine-treated mice by Western blot analysis. The expression of proteins associated with inflammasome activation, nociceptive signaling, antioxidant defenses, and oxidative stress was analyzed, including NLRP3, phosphorylated AKT (p-AKT), HO-1, SOD-1, NQO1, and the NOX isoforms NOX1, NOX2, and NOX4. Vehicle-treated animals served as controls (*n* = 4 samples per group).

A total of 144 mice (72 males and 72 females) were used in this study.

### 2.7. Protein Expression Analysis by Western Blot

Twenty-eight days after the first injection of reserpine or vehicle, animals were euthanized by cervical dislocation. Samples from the PAG, ACC and SC (L3–L6) were rapidly dissected, and total protein extracts were obtained. Tissues were homogenized by sonication in RIPA buffer supplemented with 0.5% protease inhibitor cocktail and 1% phosphatase inhibitor cocktail (Sigma-Aldrich, St. Louis, MO, USA). Samples were then incubated for 1 h at 4 °C to facilitate protein extraction, followed by a second brief sonication (10 s). Homogenates were centrifuged at 700× *g* for 20 min at 4 °C, and the supernatants were collected. Equal amounts of protein (60 µg per lane) were mixed with 4× Laemmli buffer, denatured at 95 °C, and separated by SDS–polyacrylamide gel electrophoresis (SDS–PAGE) using 4% stacking and 12% resolving gels. Proteins were subsequently transferred onto polyvinylidene fluoride (PVDF) membranes for 105 min. Membranes were blocked for 75 min at room temperature in phosphate-buffered saline with Tween 20 (PBST) or Tris-buffered saline with Tween 20 (TBST), supplemented with either 5% non-fat dry milk or 5% bovine serum albumin, depending on the target protein.

Membranes were incubated overnight at 4 °C with the following primary antibodies: anti-NLRP3 (1:200; Adipogen Life Sciences, Epalinges, Switzerland), anti-phospho-AKT and anti-total AKT (1: 150; Cell Signaling Technology, Danvers, MA, USA), anti-HO-1 (1:200; Abclonal Technology, Woburn, MA, USA), anti-SOD-1 (1:150; Novus Biologicals, Littleton, CO, USA), anti-NQO1 (1:250; Merck, Billerica, MA, USA), anti-NOX1, anti-NOX2, and anti-NOX4 (1:150; Proteintech, Rosemont, IL, USA), and anti-glyceraldehyde-3-phosphate dehydrogenase (GAPDH; 1:5000; Merck, Billerica, MA, USA) as a loading control. Primary antibodies were diluted in the corresponding blocking buffer according to the manufacturer’s recommendations.

After incubation, membranes were washed with PBST or TBST and subsequently incubated for 1 h at room temperature with horseradish peroxidase (HRP)-conjugated anti-rabbit or anti-mouse secondary antibodies (GE Healthcare, Little Chalfont, UK). Immunoreactive bands were detected using enhanced chemiluminescence reagents (ECL kit; GE Healthcare) and visualized with a Chemidoc MP Imaging System (Bio-Rad, Hercules, CA, USA). Band intensities were quantified by densitometric analysis using ImageJ (version 1.8.0; National Institutes of Health, Bethesda, MD, USA) and normalized to GAPDH levels or to the corresponding non-phosphorylated protein forms.

### 2.8. Statistical Evaluation

Statistical analyses were conducted using IBM SPSS Statistics (version 28; IBM, Madrid, Spain) and GraphPad Prism (version 9.0; GraphPad Software, LLC, La Jolla, CA, USA). All results are presented as mean ± standard error of the mean (SEM). Data distribution and variance homogeneity were assessed using the Shapiro–Wilk and Levene’s tests, respectively.

Behavioral nociceptive responses were evaluated by three-way repeated-measures ANOVA, including treatment, sex, and time as variables. Following significant omnibus interactions, simple main-effects analyses were performed using one-way ANOVA with Sidak adjustment for multiple comparisons between groups at each time point and within each sex.

Depressive-like behaviors assessed on day 28 were analyzed using a two-way ANOVA with treatment and sex as independent factors. When appropriate, comparisons within each sex were conducted using one-way ANOVA followed by Sidak’s post hoc test.

Western blot data obtained from the PAG, ACC and SC were analyzed by two-way ANOVA, with sex and treatment considered as independent factors. When appropriate, significant effects were further analyzed using one-way ANOVA followed by Sidak’s post hoc test.

Statistical significance was established at *p* < 0.05.

## 3. Results

### 3.1. DMF and CoPP Reverse Reserpine-Induced Nociceptive Hypersensitivity in Male and Female Mice

We evaluated the effects of DMF (300 mg/kg, p.o., once daily) and CoPP (2.5 mg/kg, i.p., twice daily), administered from days 25 to 28 after the first reserpine injection, on reserpine-induced mechanical allodynia, thermal hyperalgesia, and cold allodynia in male and female mice.

**Figure 2:**
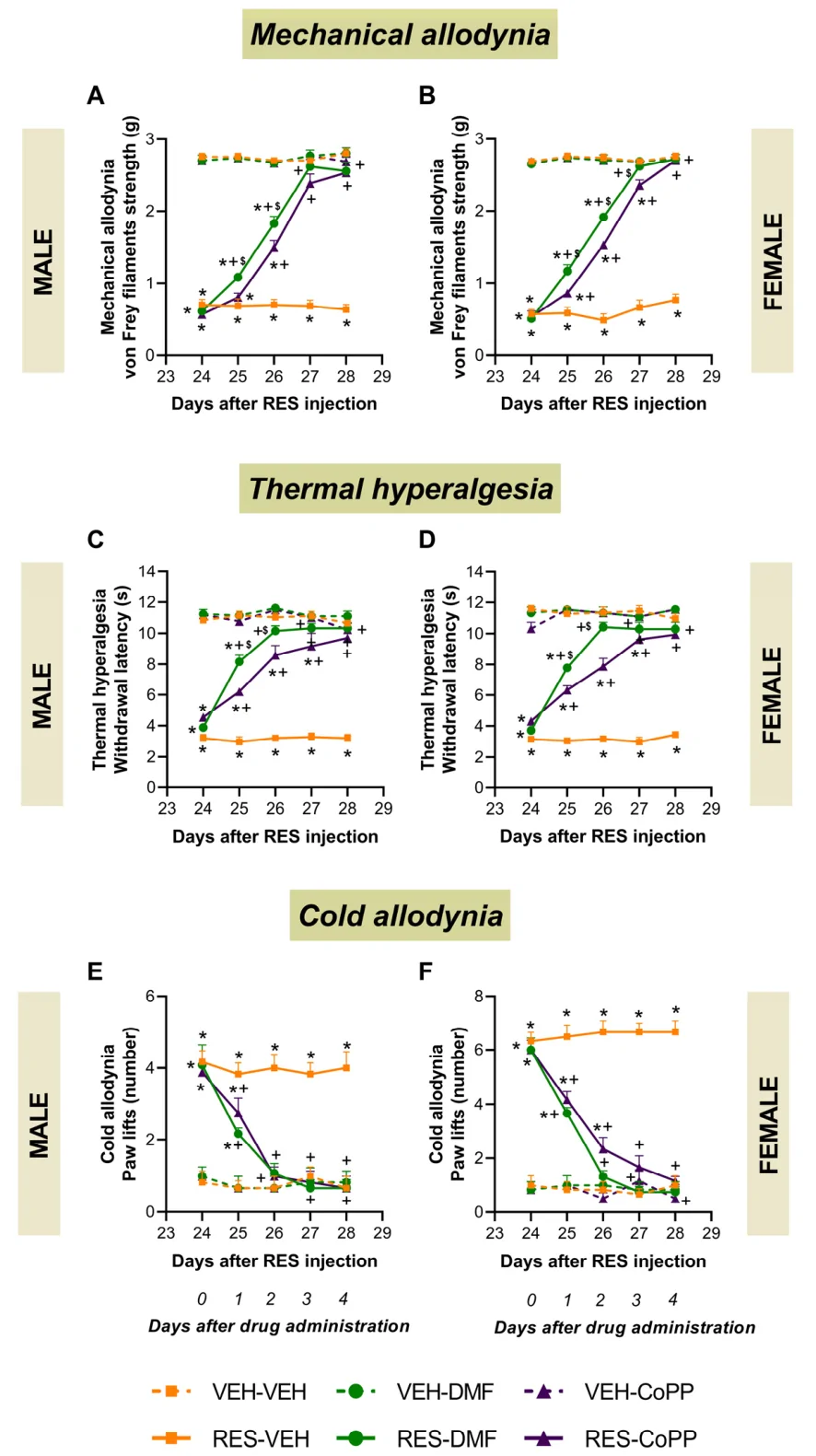
DMF and CoPP attenuate reserpine-induced nociceptive hypersensitivity in male and female mice. The effects of DMF and CoPP on mechanical allodynia in male (**A**) and female (**B**) mice, thermal hyperalgesia in male (**C**) and female (**D**) mice, and cold allodynia in male (**E**) and female (**F**) mice were evaluated in the hind paws of reserpine-injected animals. Mechanical allodynia is expressed as the von Frey withdrawal threshold (g), thermal hyperalgesia as paw withdrawal latency (s), and cold allodynia as the number of paw lifts. Symbols indicate significant differences compared with the corresponding vehicle (VEH) (*), RES–VEH (+), and RES–CoPP ($) groups at each time point (*p* < 0.05; one-way ANOVA followed by Sidak’s post hoc test). Data are presented as mean ± SEM (*n* = 6 per group). RES, reserpine; DMF, dimethyl fumarate; CoPP, cobalt protoporphyrin IX.

For mechanical allodynia, three-way repeated-measures ANOVA revealed significant main effects of treatment (*p* < 0.0001) and time (*p* < 0.0001), as well as a significant treatment × time interaction (*p* < 0.0001). Reserpine markedly reduced mechanical withdrawal thresholds, an effect that was progressively reversed by both DMF and CoPP in male and female mice. The antiallodynic effects of both treatments increased over time in both sexes, with complete reversal of mechanical allodynia achieved after three days of treatment in males (*p* < 0.0001; one-way ANOVA; Figure 2A) and after four days in females (*p* < 0.0001; one-way ANOVA; Figure 2B). Notably, DMF exhibited a faster onset of action than CoPP, producing greater antiallodynic efficacy at earlier time points, specifically after one and two days of treatment in males and after one, two, and three days in females.

In the plantar test, statistical analysis showed significant main effects of treatment (*p* < 0.0001) and time (*p* < 0.0001), as well as a significant treatment × time interaction (*p* < 0.0100). Reserpine-induced thermal hyperalgesia was completely reversed by DMF after two days of treatment in both sexes. In contrast, CoPP required a longer treatment period, with complete antihyperalgesic effects observed after four days in both male and female mice (*p* < 0.0001; Figure 2C,D). Accordingly, DMF displayed a faster antihyperalgesic onset than CoPP, producing significantly greater effects during the first two days of treatment.

Regarding cold allodynia, three-way repeated-measures ANOVA displayed significant main effects of sex (*p* < 0.0001), treatment (*p* < 0.0001), and time (*p* < 0.0001). Significant sex × treatment (*p* < 0.0001) and treatment × time (*p* < 0.0001) interactions were also detected. Both DMF and CoPP significantly reduced the number of reserpine-induced paw lifts. In males, complete reversal of cold allodynia was achieved after two days of treatment with either compound (*p* < 0.0001; Figure 2E). In females, DMF completely reversed cold allodynia after two days of treatment, whereas CoPP required three days to achieve comparable effects (*p* < 0.0001; Figure 2F).

Moreover, neither DMF nor CoPP affected nociceptive responses in vehicle-treated animals across any of the behavioral tests.

Overall, these findings demonstrate that both compounds effectively inhibited nociceptive hypersensitivity, although DMF consistently displays a faster onset of action.

### 3.2. DMF and CoPP Reverse Reserpine-Induced Depressive-like Behaviors in Male and Female Mice

Depressive-like behaviors were assessed on day 28, following four days of treatment with DMF or CoPP. As expected, reserpine administration significantly increased immobility time in both male and female mice, consistent with the development of a depressive-like phenotype in this model, as assessed by the TST and the FST.

**Figure 3:**
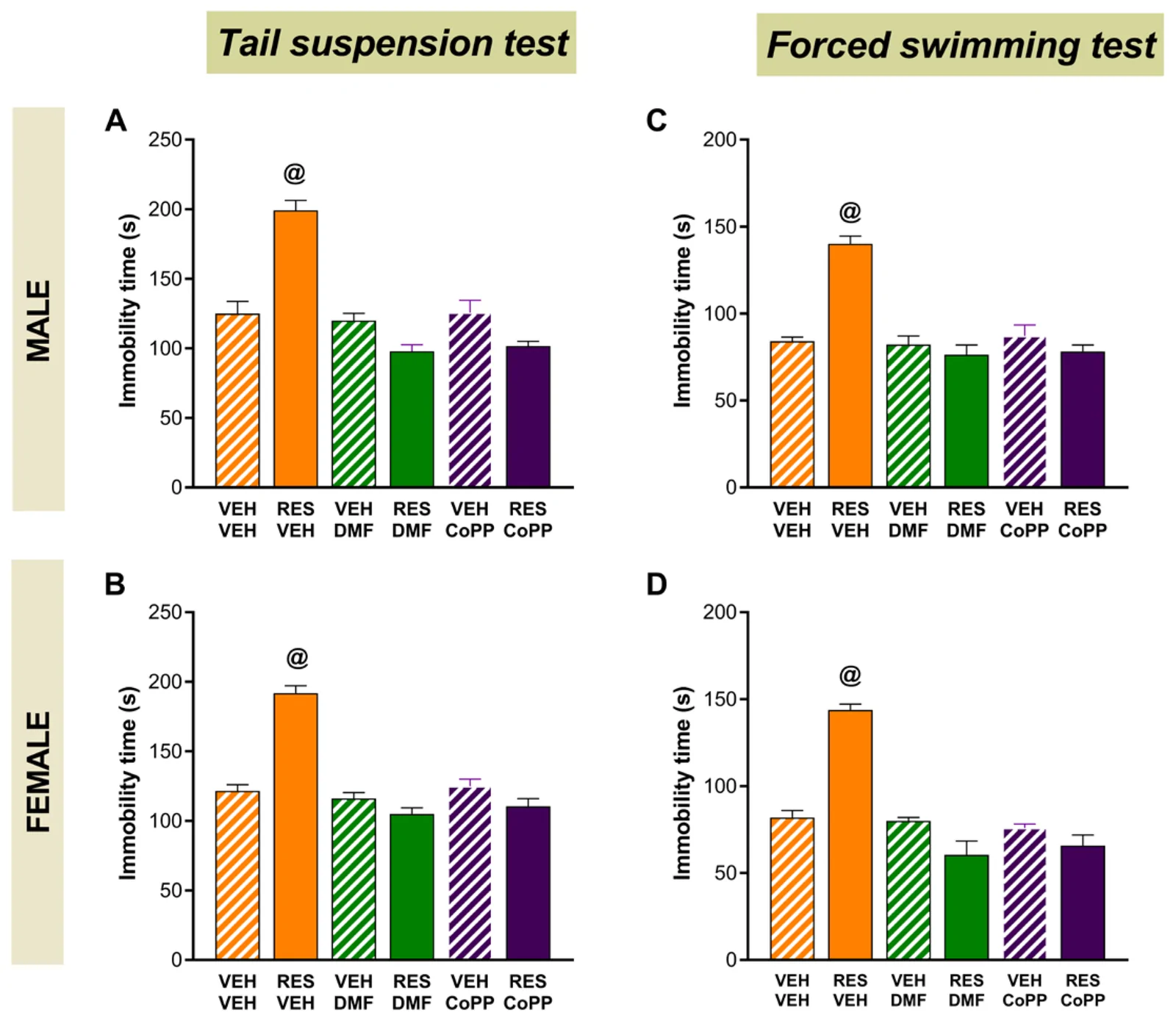
DMF and CoPP reverse reserpine-induced depressive-like behaviors in male and female mice. The effects of DMF and CoPP on immobility time were assessed using the tail suspension test (TST; (**A**,**B**)) and the forced swimming test (FST; (**C**,**D**)) in male and female mice injected with reserpine (RES) or vehicle (VEH). Behavioral evaluations were performed on day 28 following four consecutive days of treatment. The symbol (@) indicates significant differences compared with all other groups (*p* < 0.05; one-way ANOVA followed by Sidak’s post hoc test). Data are presented as mean ± SEM (*n* = 6 per group). DMF, dimethyl fumarate; CoPP, cobalt protoporphyrin IX.

Two-way ANOVA revealed a significant main effect of treatment (*p* < 0.0001), but no significant main effect of sex or treatment × sex interaction in both the TST and FST. Post hoc analyses showed that reserpine significantly increased immobility time in both sexes in the TST (*p* < 0.001; Figure 3A,B) and FST (*p* < 0.001; Figure 3C,D). Treatment with DMF and CoPP markedly reduced immobility time, restoring it to control levels in both male and female mice in both behavioral tests.

In addition, neither DMF nor CoPP significantly altered immobility time in vehicle-treated mice, suggesting that the reductions in immobility were preferentially expressed under the reserpine-induced condition rather than reflecting changes in baseline behavior.

Collectively, both treatments effectively reversed reserpine-induced depressive-like behaviors, with comparable effectiveness in male and female mice.

### 3.3. DMF and CoPP Differentially Modulate Inflammatory and Redox-Related Proteins in the PAG

**Figure 4:**
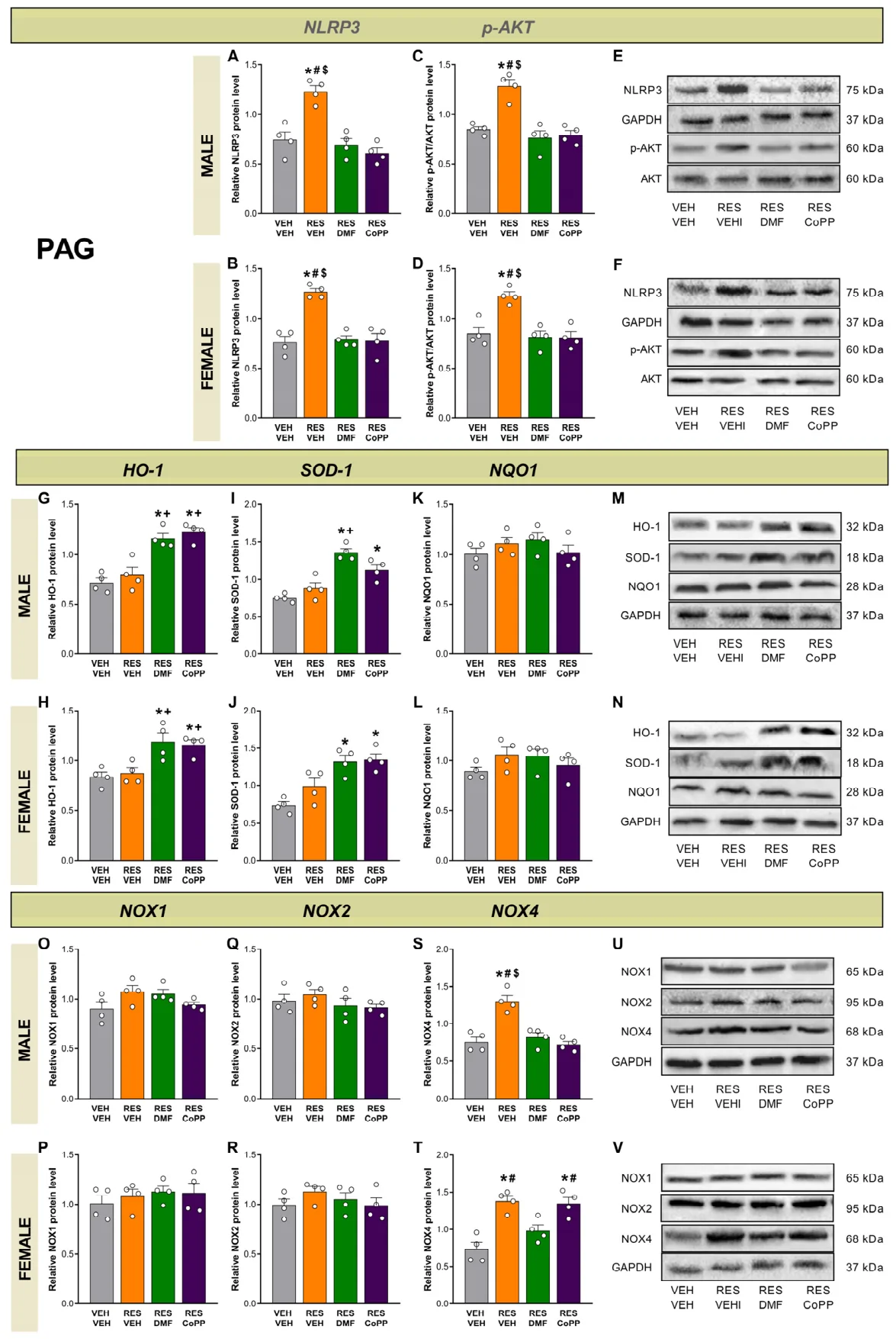
Effects of DMF and CoPP on NLRP3 expression, AKT phosphorylation, antioxidant-related enzymes, and NOX isoforms in the periaqueductal gray (PAG) of male and female reserpine-treated mice. NLRP3 protein levels (**A**,**B**) and AKT phosphorylation, expressed as the p-AKT/AKT ratio (**C**,**D**), were evaluated in the PAG of male (**A**,**C**) and female (**B**,**D**) mice treated with vehicle (VEH), reserpine plus vehicle (RES–VEH), reserpine plus dimethyl fumarate (RES–DMF), or reserpine plus cobalt protoporphyrin IX (RES–CoPP). The protein levels of heme oxygenase-1 (HO-1; (**G**,**H**)), superoxide dismutase-1 (SOD-1; (**I**,**J**)), NAD(P)H quinone dehydrogenase 1 (NQO1; (**K**,**L**)), NOX1 (**O**,**P**), NOX2 (**Q**,**R**), and NOX4 (**S**,**T**) were also evaluated in male and female mice. Representative immunoblots of NLRP3, GAPDH, p-AKT, and total AKT from male (**E**) and female (**F**) mice; HO-1, SOD-1, NQO1, and GAPDH from male (**M**) and female (**N**) mice; and NOX1, NOX2, NOX4, and GAPDH from male (**U**) and female (**V**) mice are shown. Protein levels were normalized to GAPDH, except for p-AKT, which was normalized to total AKT. Within each sex, symbols indicate significant differences compared with the corresponding VEH–VEH (*), RES–VEH (+), RES–DMF (#) or RES–CoPP ($) group (*p* < 0.05; one-way ANOVA followed by Sidak’s multiple-comparisons test). Tissue samples were collected after four days of treatment, on day 28 after reserpine administration. Data are presented as the mean ± SEM, with white circles representing individual data points (*n* = 4 independent samples per group).

Given the key role of the PAG in descending pain modulation, we investigated whether DMF and CoPP modified reserpine-induced alterations in proteins associated with NLRP3 signaling, AKT phosphorylation, antioxidant responses, and NOX-dependent oxidative processes in this brain region (Figure 4).

Two-way ANOVA revealed a significant main effect of treatment on NLRP3 (*p* < 0.0001) and p-AKT expression (*p* < 0.0001). No significant main effect of sex or treatment × sex interaction was detected for either variable. Reserpine significantly increased NLRP3 expression in male (*p* < 0.0001 vs. VEH–VEH; Figure 4A) and female mice (*p* < 0.0048 vs. VEH–VEH; Figure 4B). Similarly, reserpine increased AKT phosphorylation, expressed in males (*p* < 0.0017 vs. VEH–VEH; Figure 4C) and females (*p* < 0.0014 vs. VEH–VEH; Figure 4D). In both sexes, DMF and CoPP significantly reduced NLRP3 and p-AKT expression relative to the corresponding RES–VEH groups. Values in the DMF- and CoPP-treated groups did not differ significantly from those observed in VEH–VEH mice.

The antioxidant response in the PAG was subsequently evaluated by measuring HO-1, SOD-1, and NQO1 expression (Figure 4G–L). Two-way ANOVA revealed a significant main effect of treatment on HO-1 and SOD-1 expression (*p* < 0.0001), whereas no significant treatment effect was detected for NQO1. No significant main effect of sex or treatment × sex interaction was observed for these proteins.

Reserpine alone did not significantly alter HO-1 expression in either sex. DMF and CoPP significantly increased HO-1 expression relative to both the VEH–VEH and RES–VEH groups in male (*p* < 0.0015; Figure 4G) and female mice (*p* < 0.0022; Figure 4H). Reserpine alone also did not significantly alter SOD-1 expression. In males, DMF increased SOD-1 expression relative to both VEH–VEH and RES–VEH, whereas CoPP increased SOD-1 expression relative to VEH–VEH only (*p* < 0.0001; Figure 4I). In females, both DMF and CoPP increased SOD-1 expression relative to VEH–VEH (Figure 4J). NQO1 expression remained unchanged across all experimental groups in both male and female mice (Figure 4K,L).

Given the central role of NOX in ROS production, we next examined the expression of the NOX isoforms NOX1, NOX2, and NOX4 (Figure 4O–T). Two-way ANOVA revealed a significant main effect of treatment (*p* < 0.0001), sex (*p* < 0.0001) and a treatment × sex interaction (*p* < 0.0001) on NOX4 expression. Neither reserpine administration nor treatment with DMF or CoPP significantly altered NOX1 or NOX2 expression in either sex (Figure 4O–R).

Reserpine significantly increased NOX4 expression relative to VEH–VEH in male (*p* < 0.0264; Figure 4S) and female mice (*p* < 0.0116; Figure 4T). DMF effectively counteracted this increase in both sexes, whereas CoPP reduced NOX4 expression only in males.

Overall, these results identify the PAG as a brain region exhibiting broad molecular responsiveness to DMF and CoPP. Both treatments counteracted reserpine-induced alterations associated with inflammasome signaling and AKT activation while enhancing antioxidant defenses in both sexes. In contrast, their effects on NOX4 expression were treatment- and sex-dependent.

### 3.4. DMF and CoPP Elicit a Selective Molecular Response in the ACC

**Figure 5:**
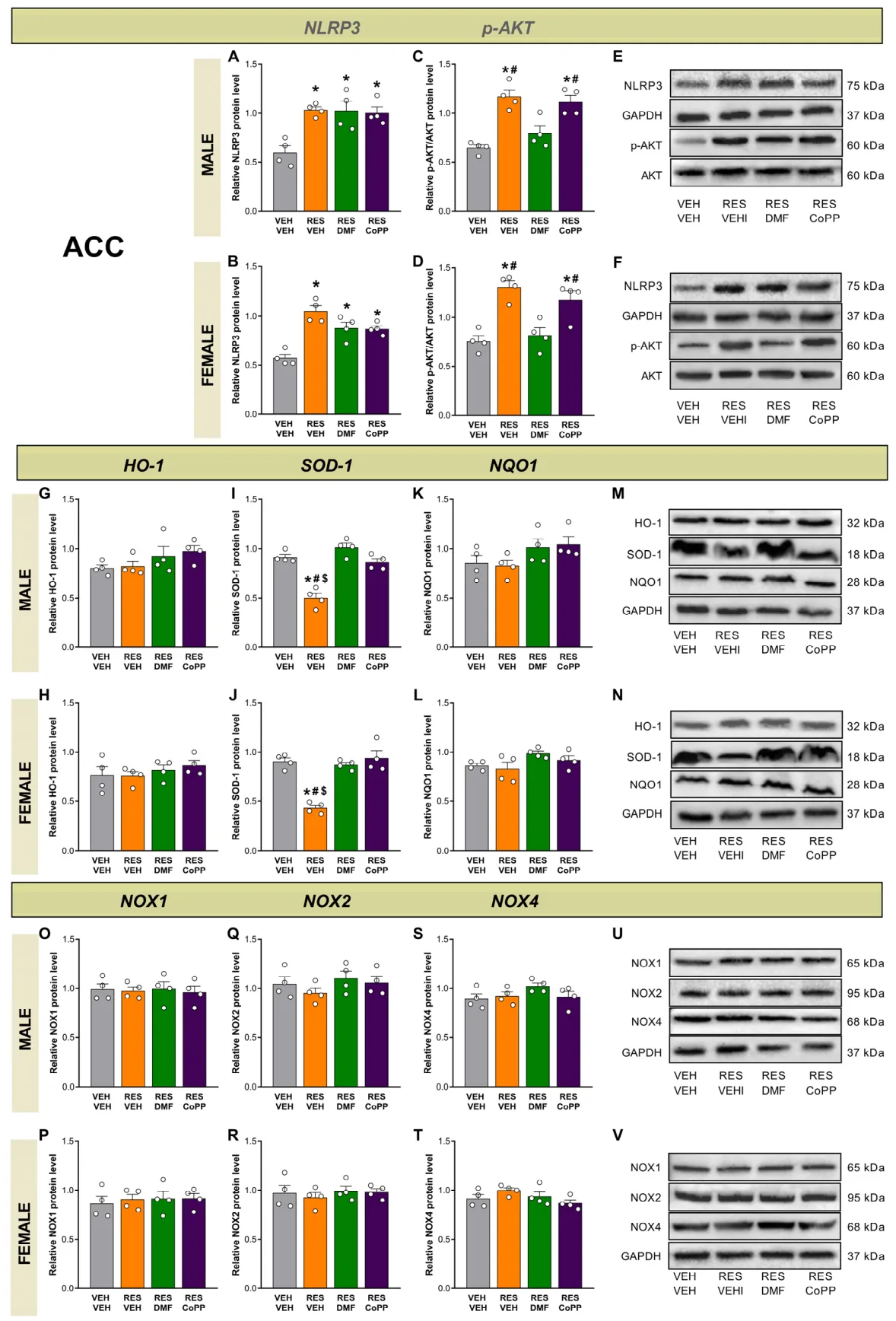
Effects of DMF and CoPP on NLRP3 expression, AKT phosphorylation, antioxidant-related enzymes, and NOX isoforms in the anterior cingulate cortex (ACC) of male and female mice. NLRP3 protein levels (**A**,**B**) and AKT phosphorylation, expressed as the p-AKT/AKT ratio (**C**,**D**), were evaluated in the ACC of male (**A**,**C**) and female (**B**,**D**) mice assigned to the vehicle plus vehicle (VEH–VEH), reserpine plus vehicle (RES–VEH), reserpine plus dimethyl fumarate (RES–DMF), or reserpine plus cobalt protoporphyrin IX (RES–CoPP) groups. The protein levels of heme oxygenase-1 (HO-1; (**G**,**H**)), superoxide dismutase-1 (SOD-1; (**I**,**J**)), NAD(P)H quinone dehydrogenase 1 (NQO1; (**K**,**L**)), NOX1 (**O**,**P**), NOX2 (**Q**,**R**), and NOX4 (**S**,**T**) were also evaluated in male and female mice. Representative immunoblots of NLRP3, GAPDH, p-AKT, and total AKT from male (**E**) and female (**F**) mice; HO-1, SOD-1, NQO1, and GAPDH from male (**M**) and female (**N**) mice; and NOX1, NOX2, NOX4, and GAPDH from male (**U**) and female (**V**) mice are shown. Protein levels were normalized to GAPDH, except for p-AKT, which was normalized to total AKT. Within each sex, symbols indicate significant differences compared with the corresponding VEH–VEH (*), RES–DMF (#), or RES–CoPP ($) group (*p* < 0.05; one-way ANOVA followed by Sidak’s multiple-comparisons test). Tissue samples were collected after four days of treatment, on day 28 after reserpine administration. Data are presented as the mean ± SEM, with white circles representing individual data points (*n* = 4 independent samples per group).

To determine whether the molecular modulation observed in the PAG extended to another supraspinal region involved in the affective and cognitive dimensions of pain, we next analyzed the ACC. Compared with the PAG, the ACC displayed a more restricted molecular response to DMF and CoPP treatment (Figure 5).

Two-way ANOVA revealed a significant main effect of treatment on both NLRP3 expression (*p* < 0.0001) and AKT phosphorylation (*p* < 0.0001), whereas neither the main effect of sex nor the treatment × sex interaction reached statistical significance.

Post hoc analyses showed that reserpine administration significantly increased NLRP3 expression in both male (*p* < 0.0083 vs. VEH–VEH; Figure 5A) and female mice (*p* < 0.0001 vs. VEH–VEH; Figure 5B). Neither DMF nor CoPP significantly modified NLRP3 expression, and protein levels remained significantly higher than those observed in VEH–VEH mice in both sexes. Similarly, reserpine significantly increased AKT phosphorylation in male (*p* < 0.0106 vs. VEH–VEH; Figure 5C) and female mice (*p* < 0.0036 vs. VEH–VEH; Figure 5D). DMF normalized the increased levels of p-AKT expression in both sexes, whereas CoPP-treated mice continued to exhibit significantly elevated p-AKT levels. These data suggest a differential effect of DMF and CoPP on AKT signaling in the ACC.

The antioxidant response in the ACC was subsequently evaluated by measuring HO-1, SOD-1, and NQO1 expression (Figure 5G–L). Two-way ANOVA revealed a significant main effect of treatment on SOD-1 expression (*p* < 0.0001), whereas no significant treatment effect was detected for HO-1 or NQO1. No significant main effect of sex or treatment × sex interaction was observed for any of these proteins.

Post hoc analyses showed that reserpine significantly decreased SOD-1 expression in both male (*p* < 0.0030 vs. VEH–VEH; Figure 5I) and female mice (*p* < 0.0001 vs. VEH–VEH; Figure 5J). Treatment with either DMF or CoPP normalized this reduction in both sexes. In contrast, neither HO-1 nor NQO1 expression was significantly altered by reserpine administration or by subsequent treatment with DMF or CoPP (Figure 5G,H,K,L).

To further characterize the oxidative profile of the ACC, the expression of the NOX isoforms NOX1, NOX2, and NOX4 was analyzed (Figure 5O–T). Two-way ANOVA revealed no significant main effects of treatment or sex and no significant treatment × sex interactions for any of the NOX isoforms. Consistent with these results, neither reserpine administration nor treatment with DMF or CoPP significantly altered the expression of NOX1, NOX2, or NOX4 in either male or female mice (Figure 5O–T).

Overall, the ACC exhibited a more selective molecular response than the PAG, characterized by the recovery of SOD-1 expression and the treatment-specific regulation of AKT signaling. These outcomes support the existence of region-specific molecular responses to DMF and CoPP within supraspinal pain/emotive-processing circuits.

### 3.5. DMF and CoPP Differentially Modulate Inflammatory and Redox Signaling in the SC

**Figure 6:**
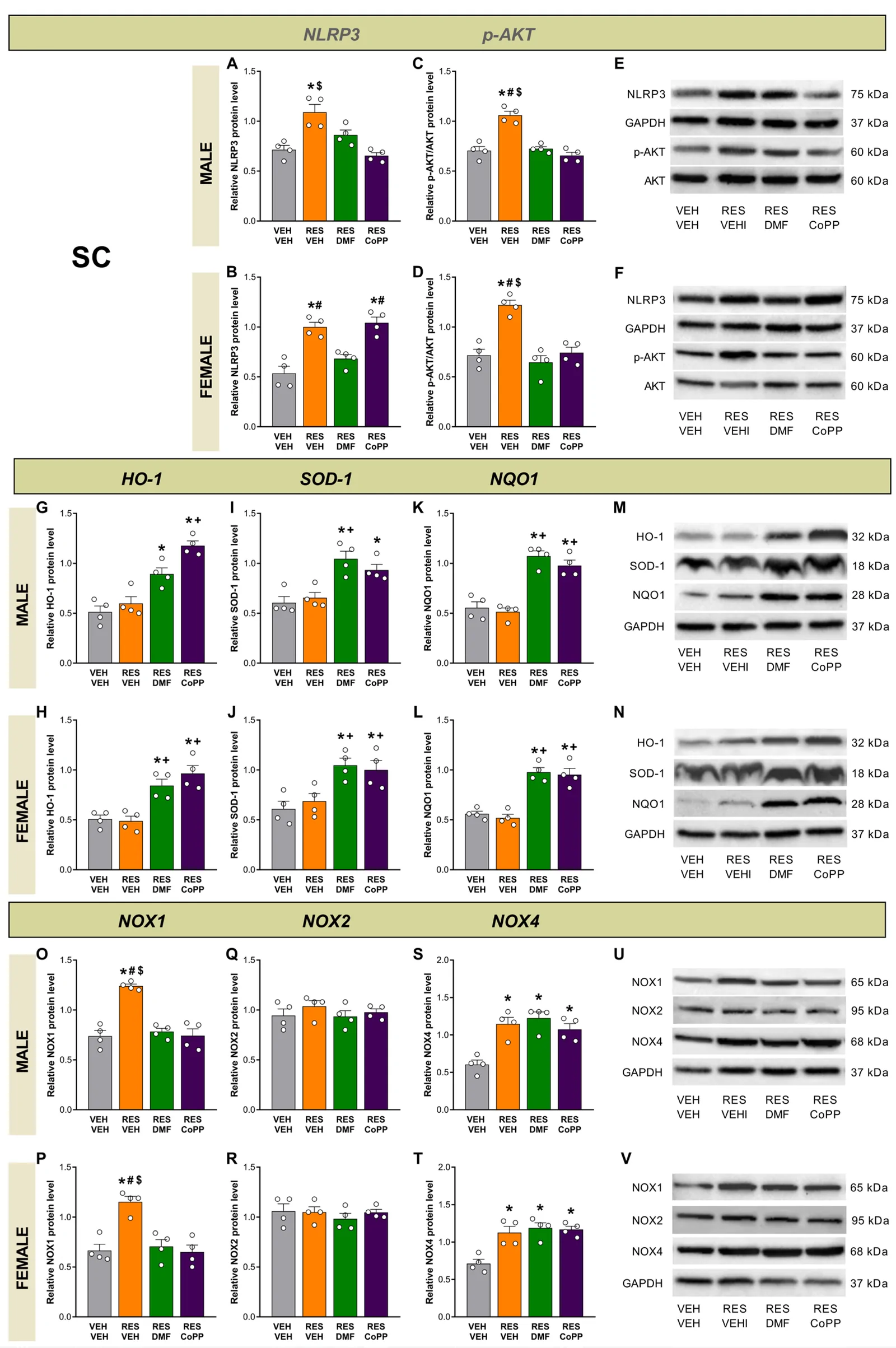
Effects of DMF and CoPP on NLRP3 expression, AKT phosphorylation, antioxidant-related enzymes, and NOX isoforms in the spinal cord (SC) of male and female mice. NLRP3 protein levels (**A**,**B**) and AKT phosphorylation, expressed as the p-AKT/AKT ratio (**C**,**D**), were evaluated in the SC of male (**A**,**C**) and female (**B**,**D**) mice assigned to the vehicle plus vehicle (VEH–VEH), reserpine plus vehicle (RES–VEH), reserpine plus dimethyl fumarate (RES–DMF), or reserpine plus cobalt protoporphyrin IX (RES–CoPP) groups. The protein levels of heme oxygenase-1 (HO-1; (**G**,**H**)), superoxide dismutase-1 (SOD-1; (**I**,**J**)), NAD(P)H quinone dehydrogenase 1 (NQO1; (**K**,**L**)), NOX1 (**O**,**P**), NOX2 (**Q**,**R**), and NOX4 (**S**,**T**) were also evaluated in male and female mice. Representative immunoblots of NLRP3, GAPDH, p-AKT, and total AKT from male (**E**) and female (**F**) mice; HO-1, SOD-1, NQO1, and GAPDH from male (**M**) and female (**N**) mice; and NOX1, NOX2, NOX4, and GAPDH from male (**U**) and female (**V**) mice are shown. Protein levels were normalized to GAPDH, except for p-AKT, which was normalized to total AKT. Within each sex, symbols indicate significant differences compared with the corresponding VEH–VEH (*), RES–VEH (+), RES–DMF (#) or RES–CoPP ($) group (*p* < 0.05; one-way ANOVA followed by Sidak’s multiple-comparisons test). Tissue samples were collected after four days of treatment, on day 28 after reserpine administration. Data are presented as the mean ± SEM, with white circles representing individual data points (*n* = 4 independent samples per group).

Finally, to establish whether the molecular alterations observed in supraspinal pain-processing regions extended to the SC, we analyzed the expression of key proteins involved in inflammatory signaling, nociceptive processing, and redox homeostasis. Compared with the PAG and ACC, the SC exhibited a distinct molecular profile, further supporting region-specific molecular responses to DMF and CoPP (Figure 6).

Unlike the PAG and ACC, the SC exhibited clear sex-dependent regulation of NLRP3 expression. Analysis of NLRP3 expression and AKT phosphorylation revealed a significant main effect of treatment for both proteins (*p* < 0.0001). A significant treatment × sex interaction was detected for NLRP3 expression (*p* < 0.0001), whereas no significant main effect of sex was observed for either NLRP3 or p-AKT expression.

Results showed that reserpine administration significantly increased NLRP3 expression in both male (*p* < 0.0036 vs. VEH–VEH; Figure 6A) and female mice (*p* < 0.0004 vs. VEH–VEH; Figure 6B). In males, CoPP significantly reduced NLRP3 expression relative to the corresponding RES–VEH group, whereas the decrease observed following DMF treatment did not reach statistical significance (Figure 6A). In females, DMF reduced NLRP3 expression to levels that no longer differed significantly from those of the corresponding VEH–VEH group, whereas NLRP3 expression remained significantly elevated following CoPP treatment. Moreover, NLRP3 expression was significantly lower in RES–DMF mice than in both the RES–VEH and RES–CoPP groups (Figure 6B). These data indicate that NLRP3 expression in the SC was regulated in a sex- and treatment-dependent manner.

Reserpine administration also significantly increased the p-AKT levels in both male and female mice compared with the corresponding VEH–VEH groups (Figure 6C,D). In contrast to the differential regulation of NLRP3, both DMF and CoPP significantly reduced the reserpine-induced increase in AKT phosphorylation in males (*p* < 0.0016) and females (*p* < 0.0007), indicating that both compounds effectively attenuated reserpine-induced AKT activation in the SC.

Regarding the expression of the antioxidant enzymes HO-1, SOD-1, and NQO1 in the SC of male and female mice (Figure 6G–L), two-way ANOVA revealed a significant effect of treatment on the expression of all three proteins (*p* < 0.0001), a significant effect of sex for HO-1 (*p* < 0.033), whereas neither sex nor the treatment × sex interaction significantly affected SOD-1 and NQO1.

Treatment with DMF or CoPP significantly increased HO-1 expression. In male mice, CoPP significantly increased HO-1 levels compared with both the corresponding VEH–VEH and RES–VEH groups, whereas DMF significantly increased HO-1 expression only compared with the VEH–VEH group (*p* < 0.0012; Figure 6G). In female mice, both DMF and CoPP significantly increased HO-1 expression relative to the corresponding VEH–VEH and RES–VEH groups (*p* < 0.0001; Figure 6H). Reserpine administration alone did not significantly alter SOD-1 expression. In male mice, DMF significantly increased SOD-1 expression relative to both the VEH–VEH and RES–VEH groups, whereas CoPP increased SOD-1 expression relative to the VEH–VEH group only (*p* < 0.0026; Figure 6I). In female mice, both DMF and CoPP significantly increased SOD-1 expression relative to the corresponding VEH–VEH and RES–VEH groups (*p* < 0.0019; Figure 6J). Similarly, treatment with either DMF or CoPP significantly increased NQO1 expression in both sexes. In male mice, NQO1 expression was significantly higher than that observed in the corresponding VEH–VEH and RES–VEH groups (*p* < 0.0001; Figure 6K). A comparable increase was observed in female mice (*p* < 0.0005 vs. VEH–VEH and RES–VEH; Figure 6L).

A distinct pattern of NOX isoform regulation was observed in the SC (Figure 6O–T). Two-way ANOVA revealed significant main effects of treatment on NOX1 (*p* < 0.0001) and NOX4 expression (*p* < 0.0001), whereas no significant treatment effect was detected for NOX2. No significant main effects of sex or treatment × sex interactions were observed for any of the NOX isoforms analyzed.

Post hoc analyses showed that reserpine administration significantly increased NOX1 expression in both male (*p* < 0.0202 vs. VEH–VEH; Figure 6O) and female mice (*p* < 0.0021 vs. VEH–VEH; Figure 6P). Treatment with either DMF or CoPP significantly reduced NOX1 expression relative to the corresponding RES–VEH group in both sexes.

In contrast, NOX2 expression remained unchanged across all experimental groups in both male and female mice (Figure 6Q,R), indicating that neither reserpine administration nor treatment with DMF or CoPP significantly affected the expression of this NOX isoform.

Furthermore, reserpine administration significantly increased NOX4 expression in both male (*p* < 0.0003 vs. VEH–VEH; Figure 6S) and female mice (*p* < 0.0144 vs. VEH–VEH; Figure 6T). Unlike the effects observed for NOX1, NOX4 expression remained significantly elevated following treatment with either DMF or CoPP in both sexes, indicating that neither treatment attenuated the reserpine-induced increase in NOX4 expression.

Overall, these outcomes demonstrate that treatment with DMF and CoPP differentially modulates inflammatory, antioxidant, and oxidative stress-related pathways in the SC. The distinct regulation of NOX1 and NOX4 further supports isoform-specific control of NOX, revealing target-specific and, in some cases, sex-dependent molecular responses within the SC.

## 4. Discussion

The present study demonstrates that treatment with DMF and CoPP attenuates both nociceptive and depressive-like behaviors in a reserpine-induced fibromyalgia-like pain model, and that DMF produces a faster and greater antinociceptive effect than CoPP. Our findings further demonstrate that oxidative dysregulation in this model is not uniformly distributed throughout the nervous system but instead is characterized by distinct region-specific redox signatures across pain-processing structures. These signatures comprise differential alterations in antioxidant defenses, ROS-generating systems, and intracellular signaling pathways, suggesting that the molecular mechanisms underlying nociplastic pain are anatomically specialized rather than homogeneous. The behavioral improvements induced by DMF and CoPP were accompanied by the normalization of many of these region-dependent molecular alterations, suggesting a potential association between the restoration of redox homeostasis and the attenuation of both sensory and affective manifestations of fibromyalgia-like pain. Collectively, these findings suggest that region-specific redox remodeling represents an important feature of nociplastic pain pathophysiology and further support the NRF2/HO-1 pathway as a promising therapeutic target for fibromyalgia.

Repeated administration of reserpine induced mechanical allodynia, thermal hyperalgesia, cold allodynia, and depressive-like behaviors in both male and female mice. These findings are consistent with previous studies demonstrating that monoamine depletion induces widespread nociceptive hypersensitivity and mood-related disturbances that closely resemble the clinical manifestations of fibromyalgia [38,39]. Oxidative stress, mitochondrial dysfunction, neuroimmune activation, and impaired endogenous pain modulation have emerged as interconnected mechanisms contributing to the development and maintenance of the nociplastic phenotype [4,40,41]. Taken together, our findings support an association between nociplastic pain and region-specific oxidative dysregulation accompanied by alterations in neuroinflammatory signaling and neuronal plasticity. Therefore, these results provide new insight into the molecular alterations associated with fibromyalgia-like pain.

Both DMF and CoPP effectively attenuated the nociceptive hypersensitivity and depressive-like behaviors induced by reserpine, indicating that pharmacological modulation of the NRF2/HO-1 antioxidant axis ameliorates both the sensory and affective components of fibromyalgia-like pain. Notably, DMF consistently produced a faster antinociceptive response, particularly against mechanical allodynia and thermal hyperalgesia. The faster antinociceptive response produced by DMF may be related to the distinct pharmacological profiles of the two compounds. DMF is a well-established NRF2 activator, whereas CoPP is widely used as an inducer of HO-1 [20,21,24,25]. However, direct NRF2 activation and HO-1 dependency were not experimentally assessed in the present study.

These distinct pharmacological profiles were accompanied by different patterns of molecular modulation. Compared with CoPP, DMF produced a more extensive normalization of region-specific molecular alterations, including a more effective regulation of intracellular signaling pathways and ROS-generating systems in selected pain-processing regions. For example, DMF normalized NOX4 expression in the PAG of both sexes, regularized p-AKT levels in the ACC, and was more effective than CoPP in reducing spinal NLRP3 expression in female mice. In contrast, CoPP displayed a more restricted molecular profile, despite ultimately producing comparable behavioral recovery.

These observations suggest that DMF not only enhances antioxidant defenses but also promotes a broader remodeling of region-specific redox networks across pain-processing circuits, whereas CoPP mainly reinforces a downstream component of the endogenous antioxidant response. Although the present study does not establish causal relationships, this more extensive molecular remodeling may contribute to the faster therapeutic onset observed with DMF.

The therapeutic potential of NRF2 activation is supported by accumulating evidence demonstrating that DMF, sulforaphane, and other NRF2 activators attenuate nociceptive hypersensitivity and affective disturbances in inflammatory and neuropathic pain models [26,27,28,29]. Our findings extend these observations to a preclinical model of nociplastic pain and indicate that pharmacological modulation of the NRF2/HO-1 axis is effective not only in reducing pain-related hypersensitivity but also in alleviating depressive-like behaviors associated with chronic pain. This dual therapeutic profile is particularly relevant for fibromyalgia, in which pain and emotional disturbances coexist and substantially contribute to disease burden [42,43,44].

One of the most important findings of the present study is that DMF and CoPP effectively attenuated neuroinflammatory signaling and maladaptive neuronal plasticity within supraspinal pain-processing regions. The PAG exhibited the most robust molecular response to antioxidant treatment, suggesting that this brainstem nucleus is particularly responsive to restoration of redox homeostasis in supraspinal areas. Following reserpine administration, the PAG displayed increased NLRP3 expression together with enhanced AKT phosphorylation, reflecting the activation of interconnected pathways involved in neuroimmune signaling and neuronal sensitization. These observations are consistent with growing evidence implicating NLRP3 inflammasome activation and downstream kinase signaling in the development and maintenance of chronic pain states [45,46]. Given the central role of the PAG in descending pain modulation [30,31], disruption of these regulatory networks may be associated with impaired endogenous inhibitory control, thereby contributing to the maintenance of widespread nociceptive hypersensitivity. Notably, the broad normalization of these molecular alterations following DMF and CoPP treatment supports the concept that restoration of redox homeostasis may contribute to re-establish the functional integrity of descending pain-modulatory circuits.

Interestingly, treatment with DMF and CoPP was also accompanied by selective reinforcement of endogenous antioxidant defenses in the PAG. Although reserpine did not significantly alter basal HO-1, SOD-1, or NQO1 expression, both DMF and CoPP markedly increased HO-1 and SOD-1 protein levels. These findings suggest that the effects of DMF and CoPP within this key pain-modulatory nucleus may be primarily associated with the enhancement of specific antioxidant enzymes rather than with uniform activation of the entire NRF2 transcriptional program. Moreover, restoration of redox homeostasis within this region may therefore contribute not only to analgesia but also to the antidepressant effects produced by DMF and CoPP.

The molecular profile identified in the ACC differed markedly from that observed in the PAG, further supporting the concept that nociplastic pain is characterized by anatomically distinct redox responses rather than by a uniform pattern of oxidative dysregulation throughout the central nervous system. As a cortical region critically involved in the emotional, motivational, and cognitive dimensions of pain [47,48], the ACC exhibited a more selective response to both reserpine and antioxidant treatment. Specifically, reserpine increased NLRP3 expression and AKT phosphorylation, whereas DMF normalized p-AKT levels without fully regularizing NLRP3 expression, CoPP exerted only limited effects on these molecular alterations. Although causal relationships cannot be established, these results suggest that cortical neuroinflammatory and intracellular signaling pathways are regulated differently from those operating within descending pain-modulatory circuits.

Remarkably, the maintenance of elevated levels of NLRP3 despite substantial behavioral recovery suggests that complete normalization of every molecular alteration within the ACC may not be required to achieve therapeutic effectiveness. Instead, these observations raise the possibility that different pain-processing regions require distinct degrees of molecular restoration according to their specific contribution to nociplastic pain. This interpretation is consistent with the complementary roles of the PAG and ACC in pain processing, where recovery of descending inhibitory pathways may have a greater impact on nociceptive hypersensitivity, whereas cortical inflammatory alterations associated with affective processing may require longer periods to resolve.

Additional evidence of this regional specialization was obtained from the analysis of antioxidant defenses. Unlike the PAG, where reserpine did not significantly alter basal HO-1, SOD-1, or NQO1 expression, the ACC displayed a selective reduction in SOD-1 without changes in HO-1 or NQO1, indicating a preferential vulnerability of cortical circuits to superoxide-mediated oxidative stress. Furthermore, both DMF and CoPP stabilized SOD-1 expression to control levels, suggesting that reinforcement of endogenous antioxidant defenses may contribute to their beneficial effects in cortical regions.

The ACC plays a central role in the emotional and cognitive dimensions of chronic pain and has been consistently implicated in depressive symptoms associated with fibromyalgia [49]. Therefore, the normalization of AKT signaling, together with restoration of antioxidant capacity following DMF treatment, may contribute not only to reduced nociceptive processing but also to the attenuation of affective disturbances, although NLRP3 expression remained elevated.

A particularly important finding of the present study is that NOX dysregulation exhibits region-specific patterns across supraspinal and spinal pain-processing structures. Rather than exhibiting a generalized increase in ROS-generating systems, reserpine induced region- and isoform-specific alterations, with selective upregulation of NOX4 in the PAG and simultaneous overexpression of both NOX1 and NOX4 in the SC. These observations indicate that different NOX isoforms may contribute to nociplastic pain through region-dependent mechanisms.

Remarkably, the response to antioxidant treatment was equally region- and isoform-dependent. DMF completely normalized PAG NOX4 expression in both sexes, whereas CoPP produced a more limited effect, particularly in females. In contrast, both compounds prevented spinal NOX1 upregulation but failed to normalize NOX4 expression in the SC. These outcomes suggest that the responsiveness of individual NOX isoforms to DMF and CoPP treatments depends not only on the isoform itself but also on its anatomical localization.

The increased spinal NOX1 expression suggests that this isoform may contribute to the maintenance of the nociceptive responses in the reserpine model, while its reversal by DMF and CoPP treatments may help explain their antiallodynic and antihyperalgesic actions. This interpretation is consistent with previous evidence demonstrating a role for NOX1-derived ROS in pain hypersensitivity in experimental fibromyalgia [50]. Nevertheless, the present findings do not establish that normalization of NOX1 is necessary for the behavioral recovery induced by DMF or CoPP.

Conversely, although increased spinal NOX4 expression suggests a potential role of this isoform in nociception, NOX4 protein expression remained elevated despite behavioral recovery following DMF and CoPP treatment. These findings suggest that normalization of spinal NOX4 protein expression is not necessary for behavioral recovery under the experimental conditions used. Both treatments also increased spinal expression of HO-1, SOD-1, and NQO1, suggesting that they could contribute to the analgesic actions of DMF and CoPP.

Together, these outcomes support the concept that NOX1 and NOX4 fulfil complementary rather than redundant functions in nociplastic pain. Whereas our data suggest that NOX1 is more closely associated with spinal sensitization, NOX4 may participate in broader redox regulatory processes operating across both spinal and supraspinal pain-processing regions. Future studies using selective pharmacological inhibitors or genetic approaches will be required to establish the specific contribution of each NOX isoform and to determine whether their functional roles vary according to anatomical location.

The more complete normalization of PAG NOX4 expression observed after DMF treatment may also contribute to its faster therapeutic onset. Although the underlying mechanisms remain to be elucidated, these results are consistent with the more extensive molecular changes observed following DMF treatment, which not only enhanced endogenous antioxidant defenses but may also have limited ROS-generating pathways in a region-dependent manner. This dual mechanism could represent an important therapeutic advantage in chronic pain conditions characterized by persistent oxidative stress [15,16,49].

In contrast to NOX1 and NOX4, NOX2 expression remained unchanged in all analyzed regions. This observation agrees with previous studies indicating that NOX2 predominantly contributes to acute inflammatory responses, whereas sustained oxidative stress during chronic pain appears to depend more heavily on NOX1- and NOX4-mediated signaling [15].

Our data also showed that both DMF and CoPP inhibited AKT phosphorylation in the SC, an effect that may contribute to the attenuation of reserpine-induced nociceptive responses. These data are consistent with previous work in the same model showing that hydrogen-rich water alleviated fibromyalgia-like behaviors while reducing reserpine-induced NLRP3 upregulation in the SC [51]. These findings further support the contribution of coordinated antioxidant, anti-inflammatory, and intracellular signaling mechanisms to the modulation of nociplastic pain [52,53].

In summary, these data suggest that the therapeutic effects of DMF and CoPP are associated with region-specific remodeling of redox homeostasis rather than uniform suppression of oxidative stress. This coordinated molecular response highlights an association between restoration of redox balance and improvement of both nociceptive hypersensitivity and depressive-like behaviors. These findings provide a strong rationale for further preclinical and clinical evaluation of NRF2 activators as potential adjunctive therapies for fibromyalgia.

The inclusion of both sexes represents an important strength of the present study, particularly considering the marked female predominance of fibromyalgia and the growing recognition that biological sex influences pain processing and treatment responsiveness [54,55]. Although DMF and CoPP ultimately produced comparable antinociceptive and antidepressant-like effects in male and female mice, females generally required longer treatment exposure to achieve complete behavioral recovery. These outcomes indicate that biological sex primarily influences the temporal dynamics of therapeutic responses rather than the overall therapeutic effectiveness of DMF and CoPP.

The mechanisms underlying these differences are likely to be multifactorial. Sex hormones modulate mitochondrial function, oxidative stress, neuroimmune activation, and endogenous pain-modulatory systems [56], while growing evidence indicates that antioxidant defenses and inflammatory signaling differ between males and females during chronic pain. These biological differences may influence both the timing and the magnitude of the therapeutic responses elicited by the pharmacological targeting of the NRF2/HO-1 pathway. This interpretation is further supported by previous studies using the reserpine-induced fibromyalgia-like mouse model, which likewise identified sex-dependent differences in antioxidant pathway recruitment and in the temporal development of nociceptive hypersensitivity [51]. Importantly, the sex-dependent effects observed in the present study were limited to specific redox and inflammatory markers rather than representing a generalized molecular response.

Several limitations of the present study should nevertheless be acknowledged. Although the reserpine-induced model reproduces many clinical features of fibromyalgia, it cannot fully recapitulate the complexity, heterogeneity, and multifactorial nature of the human syndrome. Therefore, validation of these outcomes in complementary models of nociplastic pain will be necessary to strengthen their translational relevance.

In addition, although our molecular analyses demonstrate that behavioral recovery is associated with normalization of oxidative stress-, inflammasome-, and neuronal plasticity-related pathways, they do not establish causal relationships between these molecular alterations and the observed therapeutic effects. Future works combining selective NRF2 or HO-1 inhibitors, genetic approaches, and targeted modulation of NLRP3 or individual NOX isoforms will be required to define the relative contribution of each pathway.

The relatively small sample size used for the Western blot analyses (*n* = 4 independent biological samples per group) and the fact that direct NRF2 activation was not assessed in the present study should also be considered when interpreting the molecular findings. Another limitation is that the cellular origin of the molecular alterations remains unknown. Because neurons, astrocytes, microglia, and oligodendrocytes make distinct contributions to redox homeostasis, neuroinflammation, and chronic pain, future studies using cell-specific approaches, immunohistochemistry, spatial transcriptomics, or single-cell RNA sequencing will help identify the cellular mechanisms underlying the region-specific redox signatures described here.

Finally, although the present study demonstrates robust therapeutic effects following short-term treatment, the long-term efficacy and safety of sustained treatment with DMF or CoPP remain to be established. Comparative studies examining the transcriptional, metabolic, and cell-specific consequences of DMF versus CoPP treatment will also be important to distinguish the effects of global NRF2 activation from those of selective HO-1 induction and to clarify the mechanisms underlying the broader molecular effects of DMF observed in the present study.

## 5. Conclusions

The present study demonstrates that DMF and CoPP effectively reverse nociceptive hypersensitivity and depressive-like behaviors in a reserpine-induced model of fibromyalgia-like pain. These behavioral improvements were associated with restoration of redox homeostasis, attenuation of neuroinflammatory signaling, and normalization of molecular pathways involved in neuronal plasticity across pain-processing regions.

Importantly, our findings indicate that oxidative dysregulation in fibromyalgia-like pain is regionally organized rather than uniformly distributed throughout the nervous system. The identification of distinct region-specific redox signatures involving differential regulation of antioxidant defenses, ROS-generating systems, and intracellular signaling pathways provides new insight into the molecular complexity of nociplastic pain and suggests that therapeutic efficacy is associated with remodeling these specialized redox networks rather than simply suppressing oxidative stress.

Overall, these findings identify DMF and CoPP as effective pharmacological approaches for attenuating fibromyalgia-like pain and associated affective disturbances. Moreover, the faster and greater antinociceptive effects of DMF, together with its established clinical use, highlight its potential for drug repurposing and provide a strong rationale for future mechanistic and translational studies.

## Data Availability

The original contributions presented in this study are included in the article. Further inquiries can be directed to the corresponding author.
